# S100A9 Knockout Decreases the Memory Impairment and Neuropathology in Crossbreed Mice of Tg2576 and S100A9 Knockout Mice Model

**DOI:** 10.1371/journal.pone.0088924

**Published:** 2014-02-25

**Authors:** Hee Jin Kim, Keun-A Chang, Tae-Young Ha, Jeonga Kim, Sungji Ha, Ki-Young Shin, Cheil Moon, Wolfgang Nacken, Hye-Sun Kim, Yoo-Hun Suh

**Affiliations:** 1 Department of Pharmacology, College of Medicine, Neuroscience Research Institute, MRC, Seoul National University, Seoul, South Korea; 2 Korea Brain Research Institute (KBRI), Daegu, South Korea; 3 Department of Pharmacology, Gachon University of Medicine and Science, Incheon, South Korea; 4 Daegu Gyeongbuk Institute of Science and Technology (DGIST), Daegu, South Korea; 5 Institute of Molecular Virology, Centre of Molecular Biology of Inflammation, Westfälische Wilhelms University Münster, Münster, Germany; Case Western Reserve University, United States of America

## Abstract

Our previous study presented evidence that the inflammation-related S100A9 gene is significantly upregulated in the brains of Alzheimer's disease (AD) animal models and human AD patients. In addition, experiments have shown that knockdown of S100A9 expression improves cognition function in AD model mice (Tg2576), and these animals exhibit reduced amyloid plaque burden. In this study, we established a new transgenic animal model of AD by crossbreeding the Tg2576 mouse with the S100A9 knockout (KO) mouse. We observed that S100A9KO/Tg2576 (KO/Tg) mice displayed an increased spatial reference memory in the Morris water maze task and Y-maze task as well as decreased amyloid beta peptide (Aβ) neuropathology because of reduced levels of Aβ, C-terminal fragments of amyloid precursor protein (APP-CT) and phosphorylated tau and increased expression of anti-inflammatory IL-10 and also decreased expression of inflammatory IL-6 and tumor neurosis factor (TNF)-α when compared with age-matched S100A9WT/Tg2576 (WT/Tg) mice. Overall, these results suggest that S100A9 is responsible for the neurodegeneration and cognitive deficits in Tg2576 mice. The mechanism of S100A9 is able to coincide with the inflammatory process. These findings indicate that knockout of S100A9 is a potential target for the pharmacological therapy of AD.

## Introduction

The S100 protein family represents the largest sub group within the Ca^2+^ binding EF-hand superfamily [1]. As S100 proteins have diverse functions, it is no surprise that these proteins are implicated in numerous human diseases, including different types of cancer characterized by altered expression levels of S100 proteins as well as inflammatory and autoimmune diseases [1], [2]. Some S100 proteins, such as S100A6 and S100B, play a prominent role in neurodegenerative disorders, including Alzheimer's disease (AD) [1], [3]–[6].

In a recent study on the pro-inflammatory S100A8/A9 proteins, amyloid formation was formed in the aging prostate [7], and our previous study has demonstrated that S100A9 plays a prominent role in AD [8].

Inflammation, insoluble protein deposition and neuronal cell loss are important features of the AD brain. S100A9, a the member of the calcium binding S100 protein family that is also known as MRP14 or Calgranulin B, is an inflammation-associated protein that is constitutively expressed in neutrophils and inducible in numerous inflammatory cells, including macrophages, epithelial cells, and keratinocytes [9]–[11]. S100A9 plays a role in the inflammation of the AD brain; however, a detailed mechanism has not been sufficiently reported.

Neuronal degeneration, which involves synaptic and neuronal loss, and formations of intracellular neurofibrillary tangles and extracellular neuritic plaques containing amyloid beta (Aβ) peptide plays a central role in the pathogenesis of neurodegenerative diseases, particularly in AD [12]–[15]. The enzymes β- and γ-secretase generate monomeric Aβ in neurons from amyloid precursor protein (APP) [16]. Monomeric Aβ undergoes conformational transitions and forms a dimer or trimer as well as soluble high molecular weight aggregates, and it progresses to form spherical oligomers that are composed of 12 to 24 monomers. Protofibrils elongated by these oligomers become insoluble fibrils [17], [18]. Many researchers have reported that the presence of oligomeric Aβ is more strongly correlated with disease symptoms than amyloid plaques [16], [17], [19], [20]. And aggregates of Aβ have also been shown to activate microglia and induce the production of pro-inflammatory cytokines such as tumor necrosis factor (TNF)-α, Interleukins-6 (IL-6) [21] and reduced anti-inflammatory cytokine such as IL-10 [22].

It is well known that Tg2576 mice (Tg) harboring the human APP transgene with the familial AD Swedish mutation develop AD-like cerebral amyloidosis [23], [24]. Under 6 month of age, the mice have normal memory and lack neuropathology; at 6–13 months, the mice develop memory deficits without neuronal loss; and in mice older that14 months, neuritic plaques containing Aβ form [25]–[28]. There is strong evidence that Aβ is responsible for the age-related memory decline [25], [29], [30]. In addition, Tg2576 mice develop age-dependent behavioral deficits when studied using the Y-maze and Morris water maze test [25], [26], [28].

There have been many recent studies that have examined S100A9 deficiencies. For example, in one study, S100A9 deficient mice were used to confirm the expression of Interleukin-8-induced CD11b [31]. In another study, S100A9-deficient mice were used as a model to study the role of two S100 proteins in calcium and zinc metabolism in neutrophils [10]. However, these studies were not related to AD.

To assess whether S100A9 knockout rescued the cognitive deficit and neuropathology in AD animal mice, S100A9 KO mice were crossbred with Tg2576 mice. These mice allowed for the comparison of four groups of mice; wild type/wild type (WT/WT), S100A9knock out/wild type (KO/WT), wild type/Tg2576 (WT/Tg), and S100A9knock out/Tg2576 (KO/Tg). At 13 months of age, we found that KO/Tg mice showed rescued cognitive impairments compared to WT/Tg mice. We also confirmed differences in pathogenesis, particularly abundant amyloid neuritic plaques containing Aβ and phosphorylated tau proteins and inflammatory process related cytokines in 4 groups of 14-month-old S100A9 KO/Tg crossbred mice.

## Materials and Methods

### Generation of S100A9 KOxTg crossbred mice and genotyping by PCR

All animal procedures were performed following the National Institutes of Health Guidelines for the Humane Treatment of Animals, with approval from the Institutional Animal Care and Use Committee of Seoul National University (IACUC No. SNU-100611-1). Animals of only male were used in this study.

S100A9 KO mice in a C57BL/6 background were kindly provided by Wolfgang Nacken (Münster University, Germany) [31] and crossed with Tg2576 mice expressing human APP695 with the Swedish mutation (K670N/M671L) on a C57BL/6 x SJL background. Tg2576 mice were obtained from Taconic Farms (Germantown, NY) and were bred by mating male mice with C57BL/6 x SJLF1 females, as described by others [26].

To analyze the offspring, genomic DNA samples isolated from mice tails was genotyped based on the previously described method [8], [26]. Four different genotypes (WT/WT, KO/WT, WT/Tg, KO/Tg) were studied at 14 months of age (9∼11 mice per group).

### Tissue preparation

To obtain tissues for experiments, the animals were anaesthetized and immediately cardiac-perfused with PBS containing heparin. For morphological analyses, one hemisphere of the brain was fixed in a 4% paraformaldehyde solution for 24 h and embedded in paraffin. For biochemical analyses, including western blotting, enzymatic activity assays and enzyme-linked immunosorbent assays, the other half of the brain was quickly frozen on dry ice and stored at −70°C. Tissues were lysed in RIPA buffer with protease inhibitors cocktail (Roche).

### Immunohistochemistry

Sections were deparaffinized in xylene and dehydrated using graded alcohols to water. Sections were retrieved by 0.01M citric acid (pH 6.0) and blocked with 0.5% triton X-100 and 2% normal serum in TBS. Appropriate primary antibodies were incubated overnight (O/N) and were visualized using an appropriate secondary antibody. For labeling, immunohistochemistry was performed using a Vectastain avidin biotin complex (ABC) elite kit. The reaction product was detected using 3.3-diaminobenzidinetetrahydrochloride (DAB).

### Western blot

Tissues were washed with phosphate-buffered saline (PBS) and lysed in RIPA buffer with a cocktail of protease inhibitors (Roche). Proteins were separated using SDS-PAGE and transferred to a PVDF membrane. The PVDF membrane was blocked with 5% nonfat dry milk in Tris-buffered saline containing 0.05% Tween 20 (TBS-T). After 1 h of blocking, the protein blot was confirmed using appropriate antibodies at 4°C O/N and detected using a horseradish peroxidase-conjugated secondary antibody (Amersham Pharmacia). Westen blotting was detected by Gel doc system (Bio-rad) and data was analyzed using quantity one program (Bio-rad).

### Antibodies

The following primary antibodies were used: anti-Aβ mouse monoclonal antibody 6E10 (MAB5206; Chemicon), anti-mouse S100A9 and S100A8 (AF2065, AF3059; R&D systems), S100B (ab52642;Abcam), GAPDH (Abfrontier), anti-Amyloid Oligomer, Aβ, (AB9234; Millipore),p-Tau (Ser404) (sc-12952;Santa Cruz Biotech.), Phospho-PHF-tau (S202/T205, AT8) (NM1020;Pierce), Anti-PhosphoTau (S396;PHF-13) (ab24716;Abcam), Tau (C-17) (sc-1995;Santa Cruz Biotechnology), Calnexin (H-70) (sc-11397;Santa Cruz Biotechnology), and BACE (M-83) (sc-10748;Santa Cruz Biotechnology).

### ELISA

ELISAs were performed using colorimetric sandwich ELISAs kits (human Aβ_1-42_: IBL, mouse IL-10: KMC0102, Invitrogen, mouse IL-6: DY406, R&D systems, TNF-α: DY410, R&D systems) for the quantitative determination of human Aβ_1-42_ IL-10, IL-6, and TNF-α in brains. All assays were performed according to manufacturer's specific instructions. Levels of these proteins were calculated from a standard curve developed with specific OD versus serial dilutions of known concentration. Each standard and experimental sample was run in duplicate, and the results were averaged.

### Morris water maze task

The Morris water maze was performed at 13 months after birth to measure spatial reference learning and memory based on the previously described method [8]. A training session consisted of a series of three trials per day for 5 consecutive days and a single probe trial was conducted 48 h after the final training session.

### Y-maze task

Spatial memory was assessed using the Y-maze test. The apparatus consisted of a black plastic maze with three arms that intersected at 120° (60 cm long, 15 cm high, and 10 cm wide). Vertical metal poles located at the outer perimeter of the maze provided spatial cues. A mouse was placed at the end of one arm and allowed to move freely through the maze for 8 min without reinforcements, such as, food and water. The total numbers of entries into the arms, including returns to the same arms, was recorded. Alternation was defined as entry into each of the three arms consecutively. The maximum number of alternations was calculated by subtracting two from the total number of arms entered. Percent alternation was calculated by expressing actual alternations as a percentage of maximum alternations [32].

### Passive avoidance test

As described previously [33], [34], the passive avoidance test apparatus (Model PACS-30, Columbus Instruments Int.) was used to evaluate the effects of S100A9 KOxTg crossbred mice on learning and memory. The shuttle box is divided into two chambers of equal size (23.5*15.5*15.5 cm) separated by a guillotine door (6.5*4.5 cm). The light chamber is equipped and mice can enter the dark chamber through the guillotine door. Mice were initially placed in the light chamber with the door open. If the mice entered the dark compartment, the door closed automatically. Training was repeated until the mice entered the dark compartment within 30 sec (training trial). When mice entered the dark chamber, an electrical foot shock (0.3 mA) was delivered for 3 sec through the grid floor and the door was closed automatically (acquisition trial). The mice were replaced in the illuminated chamber 24 h after the acquisition trial and the latency period to enter the dark chamber was measured for 300 sec (retention trial). If a mouse did not enter the dark chamber within the cut-off time (300 sec), it was assigned a latency value of 300 sec.

### Amyloid plaques staining

Brain sections (4 µm) were deparaffinized and hydrated using a descending ethanol series. After washing in a freshly prepared alkaline alcoholic saturated sodium chloride reagent (2.5 mM NaOH in 80% reagent-grade alcohol) for 20 min at room temperature, the sections were incubated in 0.4% Congo red (W/V, Sigma) in an alkaline alcoholic saturated sodium chloride reagent (freshly prepared and filtered prior to use) for 30 min at room temperature. Sections were washed in distilled water and counterstained with hematoxylin for 1 min. Sections were rinsed using ascending grades of ethanol with a final three changes of 100% reagent-grade ethanol, cleared in xylene and cover slipped with permount (Fisher Scientific) [32]. 0.5% thioflavin-S (Thio-S) solution was used for 5 min at room temperature.

### Secretase activity test

The fluorometric assay of secretase was conducted using β- and γ-secretase activity kits (R&D systems, Inc., USA) in accordance with the protocol supplied by the manufacturer. As an enzyme source, total cortical protein lysates were tested. Quantification of substrate cleavage was assessed using a fluorometric reader (355 nm excitation, 510 nm emission).

### Statistical analysis

Data were expressed as the mean ±SEM value or as fraction of the control value ± SEM. These results were analyzed by ANOVA followed by the Tukey HSD or the LSD test (SPSS version 18). The difference was considered statistically significant for *, *p*≤0.05, **, *p*≤0.01, and ***, *p*≤0.001.

## Results

### Generation of S100A9KO/Tg2576 crossbred mice

To elucidate whether S100A9^−/−^ plays a key role in AD progression, we crossed F1 male S100A9^+/−^/Tg2576 (HT/Tg) mice with female S100A9^+/−^/WT (HT/WT) mice to generate F2 littermates with the following genotypes: S100A9^+/+^/WT (WT/WT), HT/WT, S100A9^−/−^/WT (KO/WT), S100A9^+/+^/Tg2576 (WT/Tg), HT/Tg and S100A9^−/−^/Tg2576 (KO/Tg) mice. The experimental groups include WT/WT, KO/WT, WT/Tg and KO/Tg. In Figure S1A, the genotypes of mice from 4 groups were confirmed using PCR and western blot. We successfully obtained 4 discrete groups in the F2 littermates, as shown by the PCR and western blotting data. In immunohistochemical analysis, the S100A9 protein was significantly increased in the cortex and hippocampus of WT/Tg mice brains compared with region-matched WT/WT mice brains (Figure S1B). We found that S100A9 levels in S100A9KO/Tg mice brains were decreased compared withS100A9 levels in WT/Tg mice brains.

We investigated the levels of other specific calcium-binding proteins, including Calnexin, S100A8 and S100B in the brains of S100A9 KO/Tg crossbred mice by western blot analysis. Our data indicate that there is no change in their levels among all groups (Figure S2A). We also investigated S100A8 immunopositive cells in the brains of S100A9KO/Tg2576 crossbred mice using immunohistochemistry. Here, no differences in S100A8 expression in the brains of all groups were detected (Figure S2B).

### S100A9KO/Tg2576 crossbred mice showed significant improvements in spatial reference memory

**Figure 1:**
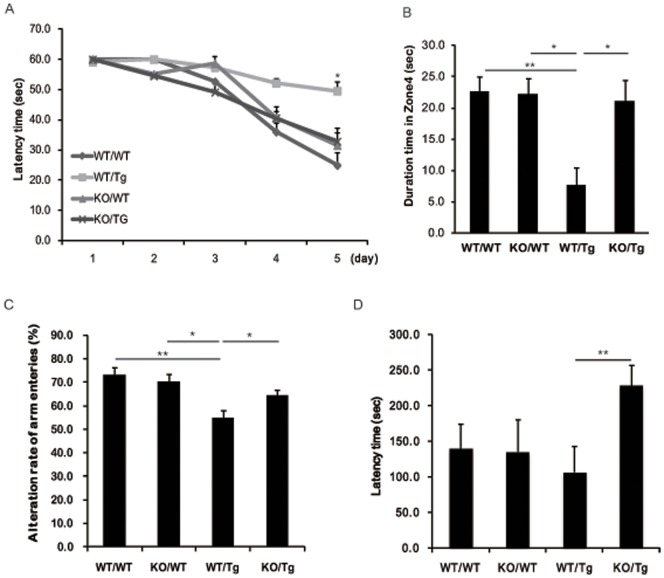
S100A9KOxTg crossbred mice showed significant improvement in spatial reference memory. We performed memory tests at the age of 13-months. (A) The Morris water maze test was performed. Training trials were conducted for 5 consecutive days. From the 5^th^ day of training trials, escape latency was significantly increased in the WT/Tg group. However, the latency was decreased in the KO/Tg group compared to the WT/Tg group.*p<0.05 by one-way ANOVA. (B) The probe test was performed 48 h after the final training session. The times that the mice of each group stayed in zones 1, 2, 3 and 4 were compared. The time spent in the platform quadrant (zone 4) was significantly decreased in the WT/Tg group. However, the KO/Tg group showed memory improvement compared to the WT/Tg group in zone 4. (C) In the Y-maze, the WT/Tg group showed a significant decrease in the alternation rate of arm entries. In the passive avoidance test, the latency time of the KO/Tg group was greatly increased. Behavior task groups were as follows: n = 7–11 per group. These results were analyzed by ANOVA followed by the LSD test *p<0.05, **p<0.01.

At 13 months of age, we evaluated learning and memory impairment in S100A9KO/Tg2576 crossbred mice using the Morris water maze task. With trainings repeated every day, WT/WT, KO/WT and KO/Tg groups found the hidden platform with less movement, and the WT/Tg group wandered with no apparent pattern (Figure 1A). On the 5^th^ day of the learning sessions, analysis of the escape latency of each group showed significant differences between the KO/Tg and WT/Tg groups (Figure 1A). We found no noticeable differences between the WT/WT and KO/Tg groups.

To confirm the memory impairment in WT/Tg mice, we performed the probe test 48 h after the final trial and recorded the duration of time spent in zone 4 without the platform. Similar to the WT/WT group, the KO/Tg mice stayed significantly longer in zone 4 than the other zones (zones 1–3) (Figure 1B). However, there was no significant difference for WT/Tg mice in terms of time spent in different zones, and no noticeable difference in the KO/WT mice (Figure 1B).

Similar results were observed in the Y-maze test. The alternation rate of arm entries in the Y-maze test was similar in the WT/WT and KO/WT groups. The alteration rate of arm entries was significantly decreased in the WT/Tg group (*P* = 0.009, F = 54.8%) but not in the KO/Tg group (*P* = 0.015, F = 64.4%; Figure 1C).These data show that knockout of S100A9 increased the spatial reference memory in KO/Tg mice.

To further examine the learning and memory function in S100A9 KO/Tg crossbred mice, we performed the passive avoidance test. As shown in Figure 1D, the latency of the KO/Tg group was shorter than the WT/Tg group in the passive avoidance test (*P = *0.003, F = 106.6). In our analysis of these behavioral test findings, the S100A9 KO/Tg crossbred mice showed an improvement in cognitive performance.

### S100A9KO/Tg2576 crossbred mice had the reduced number of amyloid plaques and decreased generation of Aβ _1-42_

Insoluble deposits of Aβ plaques are strong candidates for initiating the inflammatory response [9]. The presence of amyloid plaques in hippocampus and cortex has been confirmed using Congo red or Thioflavin-S (Thio-S) staining [1], [3].

In this study, we examined amyloid plaque load and protein levels of Aβ and CT in the brains of 14-month-old WT/WT, KO/WT, WT/Tg and KO/Tg mice using Congo red staining and western blot analysis with the 6E10 antibody, which specifically recognizes amino acids_1–17_ of Aβ.

**Figure 2:**
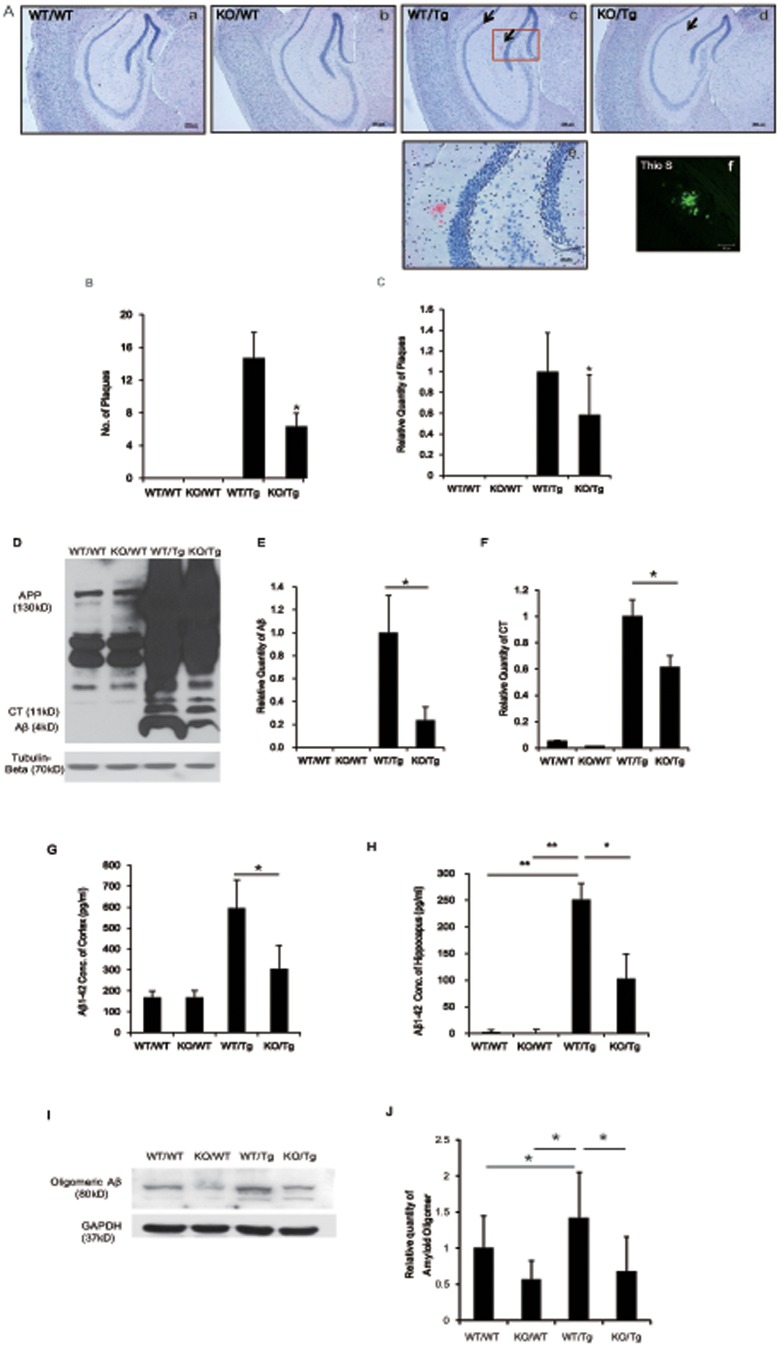
The number of amyloid plaques and amount of Aβ_1-42_ were reduced in S100A9 KO/Tg mice. After the tests, the mice brains were isolated and the brain slices were stained with Congo red for the detection of amyloid plaques. (A) Histological analysis was performed out at the age of 14-months. In the cortex, hippocampus and entorhinal cortex, amyloid plaques were detected using Congo red staining. (i)-(l) The Congo red-stained region of (c), (g) and (h). Thio-S stained region of (m). Sections are 4 µm thick. ((a) – (h) Scale bar; 200 µm, (i)-(l) scale bar; 50 µm). (B) Quantitative analysis of Congo red-stained plaque number. The number of amyloid plaques was counted in brain slices containing the hippocampal region of each group, and the average number of plaques per brain slice was calculated. In brains from the KO/Tg group, Aβ deposition was significantly reduced in the cortex and hippocampus, compared to the WT/Tg group. The total numbers of mice per group were as follows: n = 3–6 per group.*p<0.05 by one-way ANOVA. (C, D) Western blot analysis was performed with the total lysates from the cortical region and hippocampal region of brains in each group using the 6E10 antibody. Aβ and CT bands were detected and normalized by the amount of APP and GAPDH. In the WT/Tg mice brain, many Aβ and CT were produced compared to KO/Tg mice. (n = 10). (E, F) Aβ**_1-42_** levels in cortical or hippocampal brain regions in all groups were analyzed by Aβ ELISA. The levels of Aβ**_1-42_** were highly increased in the cortex and hippocampus of WT/Tg mice compared with age-matched WT/WT or WT/KO mice. Note that the levels of Aβ**_1-42_** were decreased in the cortex and hippocampus of KO/Tg mice brains compared with WT/Tg mice brains (n = 4). (G, H) Western blot analysis was performed with total lysates from the cortical region of the brains in each group using an antibody against oligomeric Aβ. In the cortex of KO/Tg mice brains, the relative quantity of oligomeric Aβ was decreased compared with WT/Tg mice. (n = 3) *p<0.05 by one-way ANOVA.

Fibrilar plaques were observed by Congo red and Thio-S staining in the cortex and hippocampus of KO/Tg and WT/Tg mice (Figure 2A and Figure S3). However, the number of amyloid plaques in KO/Tg mice was significantly reduced (from 14.7 to 6.31, *P* = 0.025; Student's *t*-test; Figure 2B). In the brains of WT/WT and KO/WT mice, no amyloid plaques were observed (Figure 2B).

Based on the Congo red staining data (Figure 2A and B), we examined protein levels of APP, APP C-terminal fragment (APP-CT) and Aβ using the 6E10 antibody. The expression of APP was no different between the WT/Tg and KO/Tg groups; however, the levels of Aβ and CT were decreased in the KO/Tg group (Figure 2D and F), which is consistent with the decreased number of amyloid plaques. The levels of Aβ and CT in the cortex of KO/Tg mice were significantly decreased (Aβ, from 1 to 0.24, *P* = 0.007; APP-CT, from1 to 0.613, *P* = 0.020; Figure 2D and F).

Using Aβ ELISAs, we confirmed the Aβ_1-42_ levels in the cortex and hippocampus of all groups. Similar to the Congo red staining and western blot results, Aβ_1-42_ in the brain of the KO/Tg group was significantly decreased by 51.17% in the cortex (from 594.84 to 304.38, *P* = 0.016; Figure 2G) and 41.03% in the hippocampus (from 250.8 to 102.9, *P* = 0.047; Figure 2H) compared to WT/Tg group.

Several studies have demonstrated that soluble Aβ oligomeric species can be extracted using saline buffers from the brain tissue of patients with AD, and the presence of soluble species is more strongly correlated with disease symptoms than amyloid plaques [16], [19], [20]. In the present study, oligomeric Aβ was detected by western blot using a specific oligomeric Aβ antibody and quantified (Figure 2I and J). In KO/Tg mice, we found a 0.47-fold decrease in oligomeric Aβ compared to the WT/Tg mice (*P* = 0.031, Figure 2J).

These data provide evidence that Aβ and CT protein levels in the brain were reduced by knockout of S100A9.

### S100A9KO/Tg2576 crossbred mice showed decreased phosphorylation of tau

Abnormal tau phosphorylation is known as a key hallmark of AD [35]–[39]. Accumulation of phosphorylated neurofilaments and phospho-tau occurs in neurites surrounded amyloid plaques in APP transgenic mice [35], [40]–[43].

**Figure 3:**
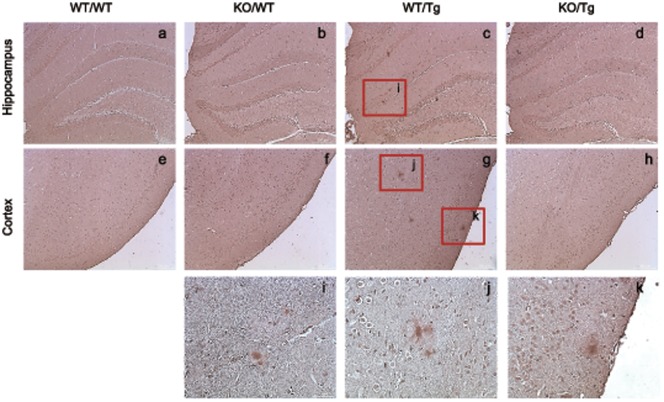
Accumulation of phosphorylated tau was detected in neurites surrounding amyloid plaques in the brain of S100A9 crossbred mice at 14 months old. Phosphorylated tau (P-tau) was detected in the hippocampus and cortex of WT/Tg and KO/Tg mice brains by immunohistochemistry. The amount of P-tau was significantly reduced in KO/Tg mice brains compared with WT/Tg mice brains. (h) – (j) are P-tau stained region of (c) and (g). Sections are 4 µm thick. ((a) – (h) Scale bar; 200 µm, (h) – (k) scale bar; 50 µm).

To determine whether S100A9 causes hyperphosphorylation of tau, we performed immunohistochemistry using brain sections with a phospho-specific tau antibody. Phosphorylated tau (P-tau), detected near Aβ plaques was reduced in the hippocampus and cortex of KO/Tg mice brains (Figure 3). And some of phosphorylated tau, such as AT8 (S202 and T205) and PHF-13 (S396), were reduced in the cortical brain of KO/Tg mice compare with WT/Tg mice (Figure S4). These data showed that the S100A9 knockout functionally recovered the pathological deficits in Tg2576 mice.

### The release of the anti-inflammatory cytokine, IL-10 was increased and the release of pro-inflammatory cytokines, IL-6 and TNF-α were decreased in 14-month-old S100A9KO/Tg2576 crossbred mice

S100 proteins, including S100A8, S100A9 and S100A12, are known to contribute to chronic inflammation [44]. Our previous study showed that treatment with siRNA for S100A9 (si-S100A9) attenuated the increase of IL-1β, TNF-α and iNOS by APP-CT. The induction of NO by APP-CT was greatly reduced by si-S100A9 treatment, which suggests that S100A9 might induce neuroinflammation by increasing intracellular Ca^2+^ levels [8]. Therefore, we focused on the mechanism related to inflammatory cytokines.

**Figure 4:**
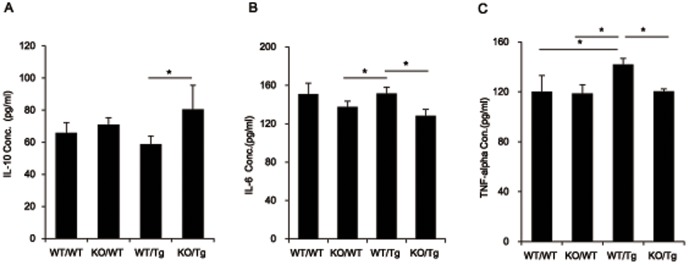
The anti-inflammatory cytokine IL-10 was significantly increased and pro-inflammatory cytokines IL-6 and TNF-α were decreased in the cortex of KO/Tg mice brains compared to WT/Tg mice brains at 14 months old. (A) The level of IL-10 was detected in the tissue lysates from the cortical region of the brain from each group by sandwich ELISA. IL-10, which is a representative anti-inflammatory cytokine, was increased in the cortex of KO/Tg mice brains compared to WT/Tg mice brains. (B) The level of IL-6 was detected in the tissue lysates from the cortical region of the brain from each group by sandwich ELISA. IL-6, which is a representative pro-inflammatory cytokine, was decreased in the cortex of KO/Tg mice brains compared to WT/Tg mice brains. (C) The level of TNF-α was detected in the tissue lysates from the cortical region of the brain from each group by sandwich ELISA. TNF-α, which is a representative pro-inflammatory cytokine, was decreased in the cortex of KO/Tg mice brains compared to WT/Tg mice brains. (n = 9–10), *p<0.05 by one-way ANOVA.

IL-10, an anti-inflammatory cytokine, has been known to have an ameliorative effect on severe inflammation by inhibiting the production of IL-12, IL-6, IFN-γ and TNF-α [22]. We confirmed the IL-10 levels in the total lysates from all groups of mice brains. In the cortex of S100A9 KO/Tg mice brains, IL-10 was increased (from 58.68 pg/ml to 80.37 pg/ml, *P* = 0.04) compared to WT/Tg mice (Figure 4A). These data indicate that a deficiency of S100A9 might inhibit severe inflammation by increasing the expression of anti-inflammatory cytokines.

IL-6 and TNF-α, which are representative pro-inflammatory cytokines, were found to be induced in reactive astrocytes surrounding beta-amyloid deposits detected in 14-month-old Tg2576 mice [45]. In KO/Tg mice brain, IL-6 was significantly decreased (from 151.4 pg/ml to 128.1 pg/ml, *P* = 0.048) compared to WT/Tg mice brain (Figure 4B). And TNF-α, was also decreased (from 136.2 pg/ml to 125.4 pg/ml, *P* = 0.035) compared to WT/Tg mice (Figure 4C). These data indicate that a deficiency of S100A9 might inhibit severe inflammation by increasing the expression of anti-inflammatory cytokines and by decreasing the expression of pro-inflammatory cytokines.

## Discussion

The S100A9 protein became the focus of current research because of its association with numerous human disorders, including acute and chronic inflammatory conditions, autoimmune diseases, cancer, atherosclerosis, cardiomyopathies and neurodegenerative diseases [46]–[48], in addition to its crucial role in normal physiological processes within cells. Recently, S100A9 has been reported to participate in the inflammation of AD pathology [7]–[9]. These studies support our hypothesis that knock out of the S100A9 gene decreased memory impairment and AD-related pathogenesis as well as neurodegeneration in an AD mice model.

To show the role of the S100A9 gene more clearly, we crossbred S100A9 KO mice and Tg2576 AD mice. We first confirmed knock-out of the S100A9 gene in the crossbred S100A9 KO and Tg2576 mice by genotyping, western blot analysis and immunohistochemistry.

Deletion of S100A9 may result in a coordinate loss of S100A8 protein because of instability of S100A8 in the absence of its binding partner (S100A9) [31], [49], [50] therefore, we measured the level of S100A8 in S100A9 KO mice. In our study, it was difficult to detect any differences in S100A8 expression from each group by western blot analysis and immunohistochemistry (Figure S2B). In addition, we investigated the expression of other specific calcium-binding proteins, including Calnexin and S100B, in S100A9 KO/Tg crossbred mice brains. We confirmed Calnexin expression, which is a calcium-binding protein localized to the endoplasmic reticulum in S100A9 KO/Tg2576 crossbred mice. However, there was no difference in Calnexin expression (Figure S2A). Overexpression of S100B in the mice brain is known to accelerate neurodegenerative disease pathology, including AD and PD [4], [51], and promote the synthesis of APP mRNA and APP in neurons, which could serve as a source of additional Aβ accumulation [52]–[55]. In S100A9 KO/Tg2576 crossbred mice, each group did not show any differences in expression of the S100B protein (Figure S2). Our data indicate that the expression levels of S100A8, S100B, and Calnexin were not changed in S100A9 KO/Tg mice.

In AD animal mice, such as the Tg2576 mice, a rapid increase of Aβ begins at from 6 months, amyloid plaques are formed after 9–12 months, and memory deficits begin after 12 months [25]–[28]. We previously reported that knockdown of the S100A9 gene significantly reduced the neuropathology, greatly improved the learning and memory deficits, and reduced the amount of Aβ and CT by decreased BACE activity [8], [56].

We crossbred Tg2576 and S100A9KO mice and investigated the behavioral and pathological characteristics of S100A9KO/Tg2576 crossbred mice. Previous results have shown that S100A9 deficiency results in attenuated spatial learning and memory behavior in tests, including the Morris water maze, passive avoidance test, and Y-maze tasks, in 14-month-old mice. In our study, we found that S100A9 is related with learning and memory impairment in the AD mice model.

Spatial memory loss was related with the appearance of Aβ aggregates [25]. Amyloid plaques and neurofibrillary tangles are believed to be the major pathological feature of AD [8], [57], [58]. Our data showed that S100A9 KO/Tg mice have a decreased amyloid plaque load and tau pathology compared to S100A9WT/Tg mice. The number of amyloid plaques and levels of monomeric and oligomeric Aβ were decreased in S100A9 KO/Tg mice. The total amount of Aβ_1-42_ was greatly decreased in KO/Tg mice compared with WT/Tg mice. These results raise the question that S100A9 may be involved in the formation of plaques and may contribute to Aβ aggregation. We previously showed that knockdown using short hairpin RNA reduced the amount of Aβ and CT by decreasing BACE activity in Tg2576 mice. In S100A9 KO/Tg mice, we did not detect significant changes in BACE activity and expression of BACE (Figure S5A and B).

In AD, tau is highly phosphorylated, which leads to the formation of neurofibrillary tangles. Phosphorylation of tau tends to provoke massive neuronal death and synaptic disruption. Therefore, we observed the level of phosphorylated tau in the brains of all mice groups. In the S100A9 KO/Tg group, tau phosphorylation was decreased. These results clearly showed beneficial pathological changes in the S100A9 KO/Tg mice.

Recent reports have shown that microglia in the brains of aged AD mice produced pro-inflammatory cytokines [34] and S100A8 and S100A9 mRNA levels were significantly increased by stimulation of IL-6 and TNF-α [59]. The anti-inflammatory cytokine IL-10 could inhibit the production of IL-6 and TNF-α [22]. Based on these studies, we observed the levels of IL-10 in the brains of all mice groups and found that IL-10 expression was higher in KO/Tg mice compared to WT/Tg mice (Figure 4A). As we expected, expression of IL-6 and TNF-α in the brains were significantly decreased in KO/Tg mice compare with to WT/Tg mice (Figure 4B and C). Therefore, the S100A9 deficiency-mediated cognitive improvements, and a reduction of AD pathology in AD models could be explained by the increased neuroprotective cytokine IL-10 and decreased inflammatory cytokines IL-6 and TNF-α.

We conclude that S100A9 KO dramatically improved the learning and memory function as well as the neuropathology of Tg2576 mice by diminishing the formation of amyloid plaques, decreasing Aβ and CT levels and up-regulating cytokines such as IL-6, IL-10 and TNF-α. Thus, we suggest that S100A9 may be a potential therapeutic candidate for inflammatory-related AD.

## Supporting Information

Figure S1 **Genotyping and protein expression of S100A9 were determined in the brains** **of WT/WT, KO/WT, WT/Tg and KO/Tg mice**. (A) For genotyping, DNA levels of S100A9 and Swedish APP were measured in each group by PCR analysis with each primer (S100A9^+/+^ for WT of S100A9 and S100A9^−/−^ for KO of S100A9; Tg2576 for Swedish form of APP). The absence of S100A9was shown in KO/WT and KO/Tg mice and the DNA band of Swedish APP was detected in WT/Tg and KO/Tg mice. Actin was used as a loading control. (B) At the age of 14-months, S100A9 expression was observed in the brain by immunohistochemisty using the anti-S100A9 antibody. In the hippocampus and cortex of mice brain, S100A9 expression was significantly reduced in KO/Tg mice compared with WT/Tg mice. Significant differences were observed in the Frontal Cortex (FC) and Parietal Cortex (PC). Sections are 4 µm thick. Scale bar; 200 µm.
(TIF)

Figure S2 **Expression of calcium binding proteins in the brains of S100A9 crossbred mice.** (A) At the age of 14-months, western-blot analysis was performed with total lysates from the cortical region of the brains in each group using anti-Calnexin, anti-S100A8 and anti-S100B antibodies. The membrane was stripped and reprobed with GAPDH to confirm equal loading. There were no noticeable differences among all groups. This is a representative blot from at least five independent experiments. (B) Immunoreactivities of S100A8 were examined in the cortex and hippocampus of 14-month-old S100A9 crossbred mice brains. There were no noticeable differences among all groups. ((a)-(l) Scale bar; 50 µm).
(TIF)

Figure S3 **Congo-red staining in the brains of S100A9 crossbred mice.** Congo-red staining was performed in the cortex of S100A9 crossbred mice brains.
(TIF)

Figure S4 **Expression of P-tau was decreased in KO/Tg mice brain.** (A) P-tau expression such as AT8 (S202, T205) and PHF-13 (S396) were decreased in KO/Tg mice brain compare with WT/Tg mice brain.
(TIF)

Figure S5 **Enzymatic activity of the β-secretase in the brains of S100A9 crossbred mice.** (A) 60 min after adding the substrate, enzymatic activity of the β-secretase from the mice brain lysates was assessed using fluorometric reaction. β-secretase activity was assessed as time passed. In S100A9 KO/Tg mice, we did not detect significant changes in BACE activity. (B) Expression of BACE showed no significant difference.
(TIF)
