# Supplementary figures and images for: S100A9 Knockout Decreases the Memory Impairment and Neuropathology in Crossbreed Mice of Tg2576 and S100A9 Knockout Mice Model
Source: PLoS One. 2014 Feb 25;9(2):e88924. doi: 10.1371/journal.pone.0088924 (PMC3934881; doi:10.1371/journal.pone.0088924)

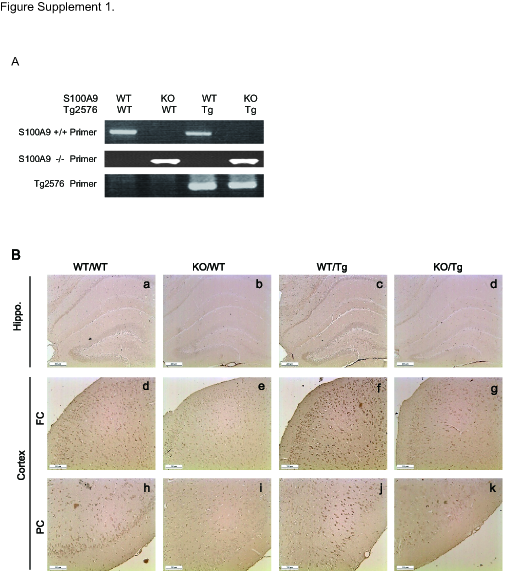

Supplement: Figure S1 — Genotyping and protein expression of S100A9 were determined in the brains of WT/WT, KO/WT, WT/Tg and KO/Tg mice. (A) For genotyping, DNA levels of S100A9 and Swedish APP were measured in each group by PCR analysis with each primer (S100A9+/+ for WT of S100A9 and S100A9−/− for KO of S100A9; Tg2576 for Swedish form of APP). The absence of S100A9was shown in KO/WT and KO/Tg mice and the DNA band of Swedish APP was detected in WT/Tg and KO/Tg mice. Actin was used as a loading control. (B) At the age of 14-months, S100A9 expression was observed in the brain by immunohistochemisty using the anti-S100A9 antibody. In the hippocampus and cortex of mice brain, S100A9 expression was significantly reduced in KO/Tg mice compared with WT/Tg mice. Significant differences were observed in the Frontal Cortex (FC) and Parietal Cortex (PC). Sections are 4 µm thick. Scale bar; 200 µm. (TIF) [file pone.0088924.s001.tif]

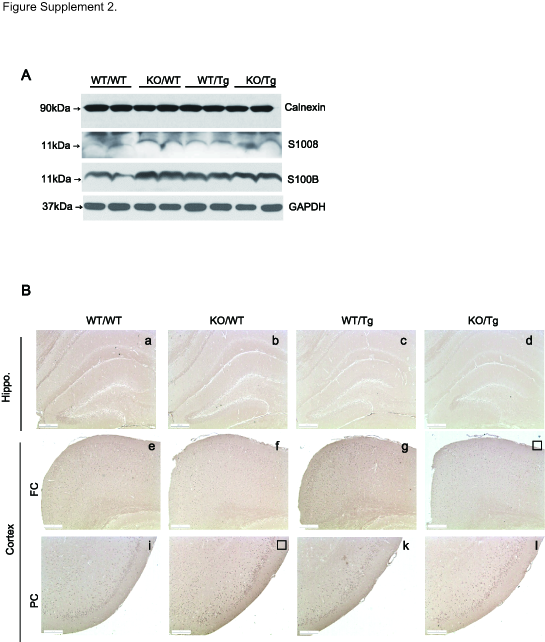

Supplement: Figure S2 — Expression of calcium binding proteins in the brains of S100A9 crossbred mice. (A) At the age of 14-months, western-blot analysis was performed with total lysates from the cortical region of the brains in each group using anti-Calnexin, anti-S100A8 and anti-S100B antibodies. The membrane was stripped and reprobed with GAPDH to confirm equal loading. There were no noticeable differences among all groups. This is a representative blot from at least five independent experiments. (B) Immunoreactivities of S100A8 were examined in the cortex and hippocampus of 14-month-old S100A9 crossbred mice brains. There were no noticeable differences among all groups. ((a)-(l) Scale bar; 50 µm). (TIF) [file pone.0088924.s002.tif]

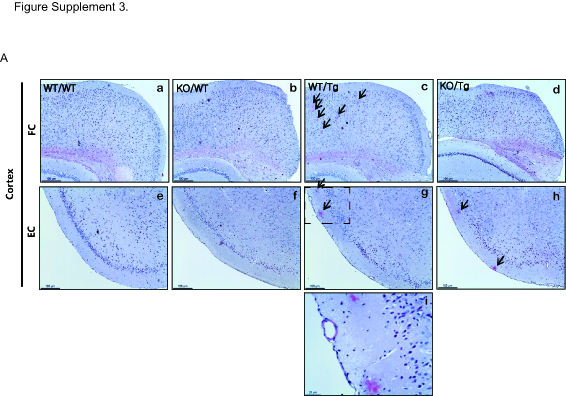

Supplement: Figure S3 — Congo-red staining in the brains of S100A9 crossbred mice. Congo-red staining was performed in the cortex of S100A9 crossbred mice brains. (TIF) [file pone.0088924.s003.tif]

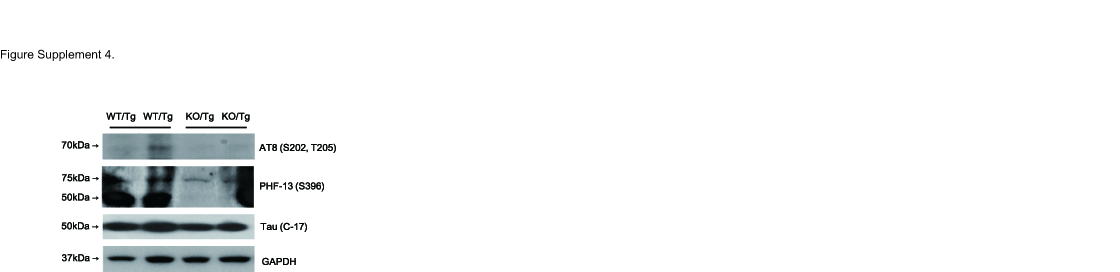

Supplement: Figure S4 — Expression of P-tau was decreased in KO/Tg mice brain. (A) P-tau expression such as AT8 (S202, T205) and PHF-13 (S396) were decreased in KO/Tg mice brain compare with WT/Tg mice brain. (TIF) [file pone.0088924.s004.tif]

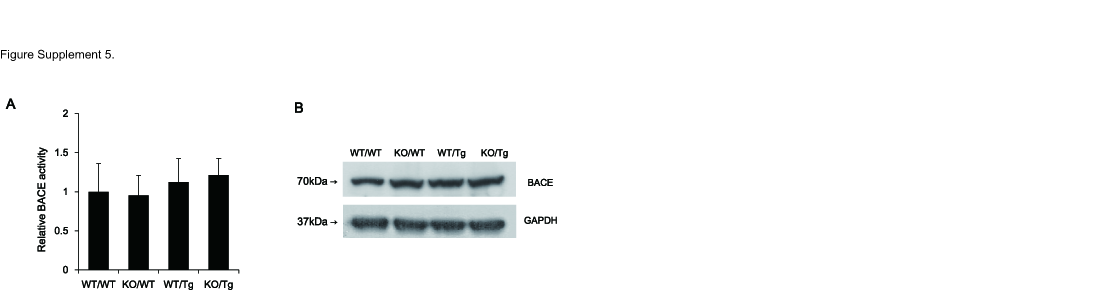

Supplement: Figure S5 — Enzymatic activity of the β-secretase in the brains of S100A9 crossbred mice. (A) 60 min after adding the substrate, enzymatic activity of the β-secretase from the mice brain lysates was assessed using fluorometric reaction. β-secretase activity was assessed as time passed. In S100A9 KO/Tg mice, we did not detect significant changes in BACE activity. (B) Expression of BACE showed no significant difference. (TIF) [file pone.0088924.s005.tif]
